# Role of apoptosis in pathogenesis and treatment of bone-related diseases

**DOI:** 10.1186/s13018-015-0152-5

**Published:** 2015-01-28

**Authors:** Samaneh Mollazadeh, Bibi Sedigheh Fazly Bazzaz, Mohammad Amin Kerachian

**Affiliations:** Biotechnology Research Center, Mashhad University of Medical Sciences, Mashhad, Iran; Biotechnology Research Center, School of Pharmacy, Mashhad University of Medical Sciences, Mashhad, Iran; Medical Genetics Research Center, Faculty of Medicine, Mashhad University of Medical Sciences, Mashhad, Iran; Department of Medical Genetics, Faculty of Medicine, Mashhad University of Medical Sciences, Mashhad, Iran

**Keywords:** Apoptosis, Bone disorder, Cell-cell interactions, Gap junction

## Abstract

In this article, bone cells and their intercellular communications have been reviewed. Gap junctions and hemichannels are the main routes of interactions in bone tissue. They play a substantial role in survival and cell death, since pro-apoptotic signals can propagate through them. Different adhesion molecules are required for apoptosis, particularly caspase family as well as noncaspase proteases. The disruption outcome of apoptosis could result in bone-related diseases such as osteonecrosis. Anti-apoptotic strategies include inhibition of caspase, poly [ADP-ribose] polymerase (PARP), and Bcl-2 proteins as well as induction of the PKB/Akt pathway and inhibitors of apoptosis (IAP) family of proteins. Thus, understanding the mechanism of apoptosis gives detailed insights of anti-apoptotic molecular targets. Based on these targets, different treatments were designed and produced such as estrogen replacement therapy, administration of different bisphosphonates, raloxifene, calcitonin, sodium fluoride, calcium, and vitamin D. As a result, new applicable drugs for treatment of related bone problems can be proposed for clinical approach especially in the early stage of diseases.

## Introduction

**Figure 1:**
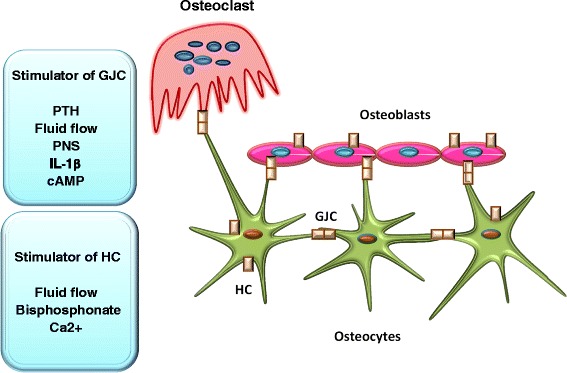
**Gap junction channels (GJC) and hemichannel (HC) in skeleton.** As it is presented in this schematic presentation, HC composing connexon placing on the body of osteocytes and osteoblasts respond to different signals which then control the transmission of different factors among are PGE2, ATP, and PNS. Also, GJC forming by contacting two HC from adjacent cells present among osteoblasts, between dendritic process of osteocytes, osteocyte-osteoblast as well as osteocyte-osteoclast. Various mechanical, hormonal, and biochemical factors affect the expression and function of GJC. Altogether, cross-talks among bone cells through these junctions regulate bone biology.

Bone as a mineralized and dynamic tissue supports and protects the rest of body [1,2]. Different kinds of stimuli affect the modeling and remodeling process in the bone tissue [3,4]. These include endocrine, paracrine, and autocrine factors, direct cell to cell communication through gap junctions [5], with greater frequency of connexin 43 (C×43), connexin 45 (C×45), and connexin 46 (C×46) [3] and cell-to-matrix communication through hemichannels [6]. Besides, coordinated interaction between bone cells plays a considerable role in bone remodeling [7]. The main bone cells are osteoblasts, osteocytes, and osteoclasts. Osteoblasts, which are responsible for maintaining skeletal architecture, originate from pluripotent mesenchymal cells [2,8]. The most abundant bone cells are osteocytes, differentiated osteoblasts, lying in the lacunar space enclosed by the bone matrix [9,10]. Osteocytes compose lacunar-canalicular network by their cytoplasmic dendrites that connect them to each other and to cells on the bone surface, important for intracellular and extracellular communication (Figure 1) [11,12]. The third main class of bone cells is osteoclast. Osteoclasts are giant multinucleated cells with abundant mitochondria, multitudinous lysosomes, and free ribosomes [2,13]. These cells differentiated from hematopoietic stem cells and are responsible for bone resorption [14,15], that is dissolving and breaking down mineral and organic substrates [16].

This article provides an overview of what is currently known about cell death in the osteoclast and osteoblast lineages and how the death of these cells may be related to clinically important bone diseases.

### Apoptosis and cell signaling

Cell death occurs under physiological and pathological conditions and mediates through three pathways as follows: apoptosis, autophagic cell death, and necrosis. In contrast to necrosis (murdered cells), in apoptosis (commit suicide) and autophagy process, cytoplasmic membranes are maintained and cell body is completely removed without any inflammation. However, if phagocytosis of the cell does not occur at the final stage of apoptosis or autophagic process, the cells are removed through secondary necrosis [12]. Apoptosis was first described by Kerr and colleagues in 1972 [17] as a physiological situation characterized by distinguishable morphological features such as nuclear changes (nuclear chromatin condensation and nuclear fragmentation), cell contraction, and losing attachment with adjacent cells. Also, this process can be governed by regulatory factors within the cell, and it can be induced or inhibited by external factors [1,18]. Additionally, apoptosis is a regulatory program which has a noteworthy role during development and growth and maintaining the skeleton [19,20]. Although insufficient apoptosis occurs in cancer or autoimmunity status, in degenerative diseases, immunodeficiency, and infertility, cell death is accelerated [21].

**Figure 2:**
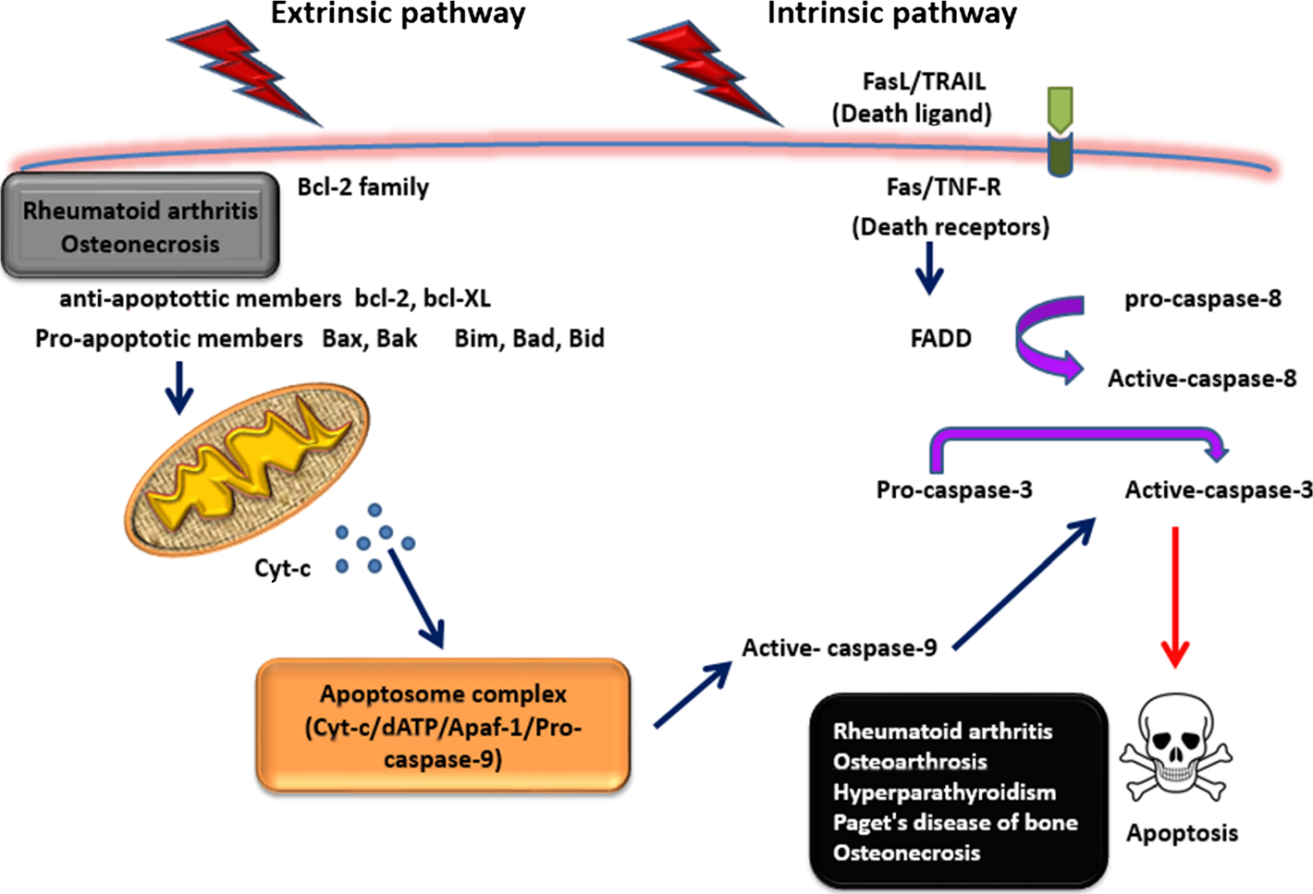
**Two apoptotic pathways are demonstrated.** Extrinsic pathway which is triggered through death receptors and intrinsic one which is caused by mitochondrial alterations. In the extrinsic pathway, binding death ligands to their receptors mainly CD95 (Fas), TNFR1, and TRAIL triggers death signal through Fas-associated death domain (FADD) which then activates caspase-8. In the intrinsic pathway, cytochrome C (Cyt-C) is released from the mitochondria and form apoptosome complex after attaching to apoptotic protease activating factor-1 (Apaf-1) followed by activating caspase-9. The activated caspase-8 and 9 (initiator) activate the effector caspases 3, 6, and 7 which then results in cell death characterized by obvious morphological and biochemical alterations. Furthermore, cell death depends on amounts of various members of Bcl2 family. For more information, read the text.

Apoptotic process triggers by different pathways: one is through mitochondrial (intrinsic pathway) and the other is established by ligand activation via death receptors (extrinsic pathway) (Figure 2) [20,22,23]. These receptors, belonging to tumor necrosis factor (TNF) receptor superfamily, include CD95 (Fas), TNF-related apoptosis-inducing ligand (TRAIL) R-1 and R-2, and TNF-R1 [21]. It has been postulated that, TRAIL as well as Fas pathways mediate osteoclast apoptosis in human [23]. The bone is to a certain extent inactive at the cellular level; thus, cell death in this tissue is a critical issue in its physiology or pathology [1]. During aging, the number of apoptotic osteoblasts and osteocytes increases, which ends up to falling in osteoblast number and bone formation [24].

The main adhesion molecules required for bone cell development and apoptosis are integrins, especially α_v_β_3_ (vibronectin receptor integrin) and α_2_β_1_ (collagen-binding integrin), selectins, caspase, and a family of transmembrane proteins containing a disintegrin and metalloprotease domain (ADAMS) [13].

Caspase activity plays a key role in osteoblast apoptosis; however, it may be also important in osteogenesis [25]. Caspase-activated DNAse (CAD), which breaks DNA down to fragments, is activated in the early phase of apoptosis. Procaspase-8 and 9 are activated by death receptor or through release of some molecules such as cytochrome C from the mitochondria. Effector caspases (caspase-3, −6, and −7) are activated after cleavage of procaspase-8 and 9. As a final point, effector caspases cleave their targets in the nucleus and cytoplasm. The release of protein-activated caspase through the mitochondria is governed by Bcl-2 family [22]. Bad, Bax, and Bid are the apoptotic members of this family while Bcl-2 and Bcl-xL are considered as anti-apoptotic components [20]. Besides, other pro-apoptotic proteins such as apoptosis-inducing factor (AIF), Smac/DIABLO, and Omi/HtrA2 are released after initial apoptotic stimuli [21]. Osteoblast apoptosis is known as a complex process which is affected by various factors including Bcl-2 family proteins, extracellular signal-regulated kinase (ERK), mitogen-activated protein kinases (MAPK), APJ/PI3-K/Akt, Janus kinase 2 (JAK2), Fas [26,27], bone morphogenetic protein (BMP), and bone matrix protein [25]. Apoptosis in osteoblasts can be triggered via physiological or pathological factors [26], i.e., osteoblasts apoptosis can be induced by oxidized lipids which then inhibit osteoblast differentiation through reactive oxygen species (ROS)-independent mechanisms. It has been shown that functional forkhead box O3 (FoxO3) in osteoblast not only reduces oxidative stress and osteoblast apoptosis but also increases osteoblasts number, bone formation rate, and mass [24]. Furthermore, matrix proteins, such as fibronectin and osteopontin are involved in osteoblast apoptosis and survival [28]. The balance between the birth of osteoblast from mesenchymal processors and the apoptotic death would determine the number of cells [18].

Another important factor in apoptosis is p53 (tumor suppressor protein) which is activated through ROS, DNA damage, and other harmful stimuli such as oncogene activation and hypoxia. Extended p53 activation leads to increased apoptosis through raised ROS levels. Also, p53 modulates ROS production through various mechanisms such as enhanced p66^Shc^ protein which affects generation of ROS in mitochondria along with converting the oxidative signals into apoptosis. During aging, phosphorylated forms of p53 and p66^Shc^ are increased in the bone as well as enhanced levels of ROS and apoptotic osteoblasts [24].

Although the life span of human osteoclasts is around 2 weeks, this time for osteoblasts is 3 months. Investigators have shown that growth factors and cytokines that affect osteoclast and osteoblast development can also stimulate their apoptosis [13].

Likewise, apoptosis in osteocytes increases with age and is related with decreased levels of receptor activator of nuclear factor kappa-B (NF-kβ) ligand (RANKL) and sclerostin protein in the bone. Accordingly, oxidative stress or other mechanisms related to aging can affect bone remodeling [24].

Since osteocytes are embedded in mineralized matrix, scavengers cannot reach them and osteocyte death causes cell disruption. Release of immunostimulatory molecules from lacunae activates macrophages producing proinflammatory cytokines such as TNF-α, interleukin (IL)-6, and IL-1. Current cytokines trigger bone loss. Besides, RANKL expression is induced via IL-6 as well as IL-1. It has been shown that apoptotic osteocytes are characterized by degraded DNA in lacunae and increased expression of specific factors, promoting osteoclastogenesis. Besides, bone resorption caused by osteocytes death is via release of intracellular content of dead osteocytes through canicular system [12]. Probably, matrix metalloproteinase (MMP)-resistant mutant of type I collagen induces osteocyte apoptosis. The osteocyte network suppresses bone formation under physiological conditions. Interestingly, osteocyte apoptosis is induced by strain causing microdamage in the bone [18]. Overall, microcracks and fatigue as well as pro-apoptotic factors trigger osteocyte death, which could activate osteoclasts. In contrast, there are some factors not only causing osteocyte survival but also osteoblast activation [10].

Altogether, apoptosis as a part of a normal physiological process could be dysregulated and causes apoptotic-related bone diseases.

### Apoptosic-related bone diseases

Extensive cell communication network made by osteocytes could detect mechanical loadings and microdamage to the bone tissue. Drugs and bone active hormones control the bone strength and integrity of this network through the regulation of osteocyte cell death [29]. Irregularities in apoptosis account for different diseases which result from either extensive or inadequate cell death [20]. Accordingly, interruption in bone remodeling, characterized by survival and apoptosis of bone tissue leads to diseases such as osteoporosis or malignant osteolysis [23].

In osteoarthrosis, hyperparathyroidism, and Paget’s disease of bone (PDB), apoptosis in osteoclasts is evident [1]. However, it has been revealed that in the osteoporotic bone, the rate of apoptosis is lower than that in the osteoarthritic bone [19]. Other bone problems mediated by osteoclast apoptosis are post-menopausal osteoporosis and rheumatoid arthritis (RA). It was revealed that Bcl-xL, an anti-apoptotic protein of the Bcl-2 family, is overexpressed in the inflamed joints of TNF transgenic mice and RA patients [30].

Osteoblast apoptosis brings about different bone problems especially osteoporosis [26]. Apoptosis in osteoblast lineage has been reported in the proximal femur particularly in the femoral head from elderly than young subjects [1]. Weinstein and his colleagues showed that glucocorticoid-induced osteonecrosis of the femoral head is not necrosis; instead, prominent apoptosis of cancellous lining cells and osteocytes occurs. Osteocyte apoptosis induced by glucocorticoid could disrupt the mechanosensory function of the osteocyte network and thus triggers a sequence of events leading to disintegration of the femoral head [31]. However, many factors are involved in etiopathogenesis of osteonecrosis such as alcohol or glucocorticoids [32,33]. We studied the role of glucocorticoids in the development of osteonecrosis [34]. Glucocorticoids induce osteonecrosis through various mechanisms, most importantly through its direct effect on bone and vascular cells causing osteoblast-osteocyte apoptosis and vascular bedding damage [35]. The effects of glucocorticoid on osteocytes lead to reduction in fluid transportation in lacunar-canalicular system, vascularity, and strength of the bone [36]. So, glucocorticoids are considered as great inhibitors of bone formation since they stimulate osteoblast apoptosis [24,35]. The mechanism by which glucocorticoids stimulate apoptosis is reported to depend on interactions between glucocorticoid receptors, dimerization of receptors and regulation of gene expression. Dexametasone (Dex), as a glucocorticoid, may increase the expression of pro-apoptotic genes inducing apoptosis [37]. Furthermore, aminobisphosphonates used for the treatment of osteoporosis and other disorders of bone resorption may induce apoptosis in osteoblasts and inhibit their differentiation [38]. Increased expression of inducible nitric oxide synthase (iNOS) in osteonecrotic patients elevates toxic levels of NO, which have deleterious effects on osteoblastogenesis followed by high amounts of apoptotic cells [32].

It was reported that increased apoptosis in myeloma bone microenvironment accounts for impaired new bone formation due to high amounts of cytokine as well as physical interaction among osteoblasts and malignant plasma cells [39]. Studies also have shown that pathogenesis of osteosarcoma is related to abnormal function of p53 and the retinoblastoma gene [1]. Altogether, osteoblast apoptosis which is to some extent influenced by lack of sex steroid, excess of glucocorticoids, or aging results in bone loss [19].

### Anti-apoptotic factors in bone loss

Anti-apoptotic procedures in the case of pathologically increased cell death are as follows: inhibition of caspase, poly [ADP-ribose] polymerase (PARP), and Bcl-2 proteins as well as induction of the PKB/Akt pathway and the inhibitor of apoptosis (IAP) family of proteins [20]. It was shown that overexpression of Bcl-2 prevents apoptosis through cytochrome C release inhibition [40]. Activation of PKB/Akt by kinases like phosphoinoside-dependent kinase 1 (PDK1) creates protection against apoptosis [41]. Also, the anti-apoptotic effect of IAP occurs by suppressing enzymatic function of caspases [42]. Various apoptotic-related diseases can be treated by inhibition of caspase activities followed by a decrease in apoptosis and organ function refining. It was reported that Casp3Inh (Z-DEVD-FMK) inhibits caspase-3 activity which could affect bone mineral density (BMD) by upregulation of transforming growth factor (TGF-β)/Smad2 signaling pathway in bone marrow stromal stem cells (BMSSCs). Furthermore, it was proved that Z-DEVD-FMK prevents osteogenic differentiation in human BMSSCs through inhibition of caspase-3 activity. Therefore, caspase-3 plays a considerable role in bone development and metabolism [25]. Besides, survival signals can be generated by intracellular kinase cascade including states, ERK, Akt, and phosphathidyl inositol-3-kinase (PI3K). Along with this, focal adhesion kinase (FAK) activation activates PI3K and/or the MAP kinase cascade, which prevents apoptosis [43]. PI3K, which is activated through different extracellular signals such as insulin and insulin-like growth factor I (IGF-I), significantly is involved in several anti-apoptotic pathways as well as enhancing proliferation by Akt/PKB. Phosphatase and tensin homologue (*PTEN*) as a tumor-suppressor gene prevents the activity of PI3K. Lack of *PTEN* increases bone mass through promoting the number of osteoblasts probably via the deceleration of apoptosis [26]. Recent studies suggest that Wnt/b-catenin activates anti-apoptotic signaling pathways that primarily act through Src/ERK and PIK3/Akt [18]. Activation of NF-kB is considered as an inhibitor of apoptosis induced by death receptor [30]. TGF-β, IGF-I, fibroblast growth factor 2 (FGF-2), and IL-6 have anti-apoptotic effects on cultured osteoblastic cells. Moreover, MMP-resistant mutant of type I collagen has the same effect on these cells [18]. IL-1 is considered as an anti-apoptotic factor which its lack results in increase osteoclast apoptosis by diminishing prostaglandins concentration or other anti-apoptotic factors. Osteoclasts apoptosis can be prevented by 1,25 (OH)2 vitamin D3 and parathyroid hormone (PTH) probably through RANKL stimulation or reduction in osteoprotegerin (OPG) expression. It was suggested that PI3K/Akt signaling pathway has a positive regulatory effect on osteoclast formation. Macrophage colony-stimulating factor (M-CSF) and RANKL stimulate the expression of anti-apoptotic gene Bcl-2 and Bcl-xL and x-linked inhibitor of apoptosis protein (XIAP). Besides, RANKL-activated NF-kB is required for osteoclast survival [43]. NF-kB, which has an anti-apoptotic effect on some cell types including osteoclasts, is activated by binding TNF to its receptor [24]. In contrast, strontium ranelate enhances osteoclast apoptosis through a calcium-sensing receptor (CaR)-dependent mechanism [44]. OPG, a TRAIL receptor, plays a key role in the inhibition of apoptosis induced by TRAIL [30]. Similar to M-CSF, IL-1, TNF-α, and IL-6, there are other cytokines which inhibit osteoclast apoptosis. Inducer factors of bone resorption inhibit osteoclast apoptosis, while their suppression stimulates the apoptosis. Estrogen, 17f-oestradiol (E2), increases the number of apoptotic osteoclasts; however, the effect of E2 on osteoclast could be reversed by a pan-specific anti-TGF antibody as well as estrogen agonist/antagonist tamoxifen. Likewise, integrins such as α_v_β_3_ integrin has a similar effect on the bone which increases osteoclast apoptosis. Other findings suggest that interactions between osteoclast matrix regulate osteoclast apoptosis [1]. The same cytokines and growth factors affect not only osteoblast and osteoclast development but also their apoptosis. In this case, IL-6 blocks apoptosis of osteoblastic cells of animal and human as well as osteoclasts and their processors. Also, it has been reported that TGF-β has an anti-apoptotic effect on osteoblast while it could increase osteoclast apoptosis [13]. Several endogenous stimuli (systemic, local, or mechanical) have anti-apoptotic effects on bone cells through C×43 or cell-to-cell communication. Similarly, anabolic and anti-catabolic inducers have anti-apoptotic effects on bone-forming cells such as the anabolic effects of PTH, activators of the Wnt signaling pathway, and mechanical stimuli [45].

### Anti-apoptotic drugs and treatment of bone loss

Bisphosphonates are anti-catabolic drugs administering in disorders including malignant osteolysis, osteoporosis, and PDB [30]. It was reported that bisphosphonate drug (alendronate) needs C×43 hemichannels to inhibit apoptosis in osteoblast through activation of src-ERK [38,45,46]. It is supposed that alendronate enters to the cell after inducing C×43 opening. Alendronate causes closure of C×43 hemichannels by phosphorylation of the C-terminal cytoplasmic domain after interaction with Src kinase, the upstream activator of ERK [47]. Besides, the current drug has the same effect on osteocytes via cytoplasmic ERK activation and influencing the canonical nuclear translocation pathway signaling cascade [3,48]. Likewise, alendronate has a prevailing effect on inhibition of osteoclastic resorption in glucocorticoid-induced bone loss [45]. Since bisphosphonate increases osteoclast apoptosis in human and rodents, it is considered as a potential treatment in PDB and post-menopausal osteoporosis [1]. As the mechanism of action of bisphosphonates in osteoblasts and osteocytes depends on C×43 hemichannels, the current compounds are considered as an anti-apoptotic pharmacological target [38,45,46]. Because sex steroid paucity as well as glucocorticoids induce bone apoptosis, protection against them preserves bone cell viability [45]. Hormones, estrogens, and androgens inhibit apoptosis in osteoblasts and osteocytes. Also, 1,25-dihydroxyvitamin D_3_ has anti-apoptotic effects on osteoblasts. Additionally, PTH and PTH-related protein (PTHrP) have the similar effects on cultured osteoblasts of rat, murine, and human via inactivation of Bad, enhanced transcription of survival genes such as Bcl-2, and cAMP-activated protein kinase A [18]. Not surprisingly, some natural compounds have therapeutic effects such as reveromycin A (RM-A). This compound, a small natural product with three carboxylic groups isolated from the genus *Streptomyces*, induces apoptosis in osteoclasts through induction of cytochrome C release and caspase 3 activation [49]. It was shown that puerarin, which is extracted from the root of a wild leguminous creeper with estrogen-like structure, has an anti-apoptotic effect on human osteoblasts by activation of ERK signaling pathway. So, it could be used as a potential drug for osteoporosis treatment [26]. Similarly, vaspin, an adipocytokine isolated from visceral white adipose tissue, prevents apoptosis on the same cells probably via activating the MAPK/ERK signaling pathway [50]. Besides, adrenomedullin (ADM), synthesized and secreted by a variety of cells, enhances the number of osteoblasts and increases bone growth. Recently, it was shown that ADM possess an osteoblastic anti-apoptotic effect through calcitonin gene-related peptide1 (CGRP1) receptor-MEK-ERK pathway [51]. Apelin, a peptide expressed by adipocyte and osteoblast, inhibits apoptosis mediated by serum deprivation in human osteoblasts through APJ/PI-3 kinase/Akt signaling pathway. It should be noted that APJ is an orphan G protein-coupled receptor and apelin is its endogenous ligand. Apelin has a protective role in glucocorticoid-induced apoptosis [52]. As mentioned earlier, glucocorticoids suppress the number of osteoblasts through reducing ERK activation by mitogens. It has been shown that vanadate (a protein tyrosine phosphatase inhibitor) could act as a survival factor since it inhibits apoptosis mediated by glucocorticoids in pre-osteoblasts and osteocytes [37]. There are lines of evidence that taurine, the most plentiful intracellular amino acid in humans, could conserve osteoblast through taurine transporter (TAUT)/ERK pathway. Moreover, the mechanism of the taurine is associated with reduction of mitochondria-dependent pathways that is decreasing the release of cytochrome C [53]. Taken together, estrogen replacement therapy (ERT), different bisphosphonates (e.g., alendronate), the selective estrogen receptor modulator (SERM), raloxifene, calcitonin, sodium fluoride, calcium, and vitamin D could be used as modulators to treat bone diseases. Androgen and estrogen inhibit apoptosis in osteoblasts and osteocytes; thus, estrogenic, androgenic, or even nonsteroidal compounds are considered as potential drugs for osteoporosis treatment [13].

## Conclusion

To sum up, bone diseases especially the intolerable ones could be treated in the early stages of the disease via therapeutic factors triggering the anti-apoptotic pathways. Based on preclinical studies, molecular routes encourage us to think about practical target achievements. However, molecular therapies should be used with more care because of their bilateral role in some cases.
